# Eosinophilic Fasciitis following Checkpoint Inhibitor Therapy with Pembrolizumab

**DOI:** 10.31138/mjr.32.4.376

**Published:** 2021-12-27

**Authors:** Evangelia Zampeli, Eleftherios Zervas

**Affiliations:** 1 Rheumatology Department, Lefkos Stavros - The Athens Clinic, Athens, Greece,; 2 7^th^ Pulmonary Department and Central Bronchoscopic Unit “Christos Gagas” NHS, Athens Chest Hospital “Sotiria”, Athens, Greece

**Keywords:** Eosinophilic fasciitis, checkpoint inhibitors, rheumatic immune-related adverse events

## Abstract

With the increasing number of indications for checkpoint inhibitor therapy in cancer patients, rheumatology specialists are often involved in the diagnosis and management of immune-related adverse events (irAEs). The most common rheumatic irAEs are arthritis, sicca syndrome, polymyalgia rheumatica, and myositis. Eosinophilic fasciitis, an already rare rheumatic disease, is a very unusual rheumatic irAE. Eosinophilic fasciitis, whether idiopathic or checkpoint inhibitor-associated, has very particular clinical symptoms and findings, such as the orange peel skin appearance and the Groove sign, which the physicians need to be aware of and recognise timely in order to diminish morbidity.

## CASE REPORT

A 61-year-old male patient, former smoker (60pack-years) with type-II diabetes since his 40s, was diagnosed sixteen months ago (April 2019) with non-small cell lung adenocarcinoma, stage IVB (left upper lobe mass 12.2 cm, lymph-node 5 and 7 up to 5 cm and left adrenal gland metastasis 1.7 cm). He started 1^st^ line chemotherapy with carboplatin (5 AUC) and pemetrexed (500mg/m^2^) every twenty-one days. He received three chemo-therapy cycles; however, restaging CT scans showed disease progression with pleural and pericardial effusion and increase in left upper lobe mass size. Due to the unsatisfactory results of chemotherapy and the tumour programmed cell death-ligand 1 (PD-L1) positivity (5%, tumour proportion score (TPS)≥ 1–49%), second-line treatment with the checkpoint inhibitor (CPI) pembrolizumab (PD-1 inhibitor, 2 mg/kg every 3 weeks) was initiated in November 2019. The patient continued treatment for twelve months with pembrolizumab with stable disease. One month before evaluation, the patient developed, without prior physical strain, progressive intense diffuse arthralgias and myalgias, accompanied by the sensation of stiffness and muscle weakness. With the suspicion of a possible immune-related adverse event (irAE) and a preliminary diagnosis of polymyalgia rheumatica, pembrolizumab was discontinued and the patient was referred to a rheumatology unit.

**Figure 1.**
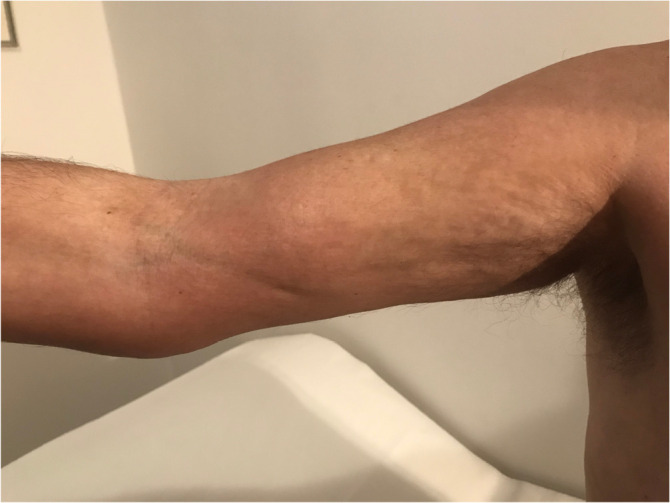
Venous furrowing (*Groove sign*) and orange peel (*peau d’ orange*) appearance is seen on the oedematous, woody skin of this patient with pembrolizumab-related eosinophilic fasciitis.

Upon presentation, skin thickening with a “woody” texture of the forearms and painful, symmetrical oedema of the lower limbs, below the knees was noted, which limited the mobility of the elbows, wrists, knees, and ankles. The fingers, trunk, and face were not affected. When the patient was asked to raise his arms, a peau d’orange skin appearance as well as a depression along the course of superficial veins (Groove sign) was seen on his forearms (**Figure 1**). Laboratory testing showed a continuously increasing absolute eosinophil count (maximum 3280/μL), elevated erythrocyte sedimentation rate (ESR, 69mm/h) and serum C-reactive protein (CRP, 55mg/L, normal value<5), diffuse hypergammaglobulinemia (γ globulins 20.8%) and high serum IgG (2060mg/dl, normal range 700–1600mg/dl). Chemistry (including muscle enzymes), glycosylated haemoglobin, and thyroid function tests were within normal levels. Autoantibodies (antinuclear antibodies, rheumatoid factors, anti-cyclic citrullinated peptide, and anti-Scl70) were negative. The patient’s symptomatology, physical examination, and laboratory findings were all suggestive of eosinophilic fasciitis (EF). EF is a scleroderma-mimicker that classically begins with oedema, erythema, and subsequent painful and symmetrical tightening of the limbs, which may give an orange peel appearance. EF may progress for weeks to months, evolving to fibrosis, hyperpigmentation, and woody appearance of the skin, which leads to flexion contractures and decreased mobility. The orange peel appearance of the skin, the presence of the Groove sign as well as the absence of sclerodactyly, telangiectasia, joint sensitivity, and Raynaud’s phenomenon distinguishes EF from scleroderma. The face and digits are typically spared.^1,2^ Peripheral eosinophilia is found in 60–90% of EF patients along with polyclonal hypergammaglobulinemia and increased inflammatory indices.^2^ Full-thickness skin biopsy including the muscle and the fascia confirms the diagnosis. Magnetic resonance imaging (MRI) of the affected limbs is also useful in the diagnosis, showing contrast-enhanced thickened fascia.^3,4^ In the case of our patient, unfortunately, neither the biopsy nor the MRI was performed. The patient refused, feeling both insecure due to the Covid-19 pandemic, but also worn out by his disease.

Other possible differential diagnoses, such as scleromyxedema (rare condition of mucinous deposition in the skin associated with the presence of a monoclonal gammopathy), scleroedema (occurring in poorly controlled diabetes, monoclonal gammopathies and after certain infections, particularly streptococcal pharyngitis), and nephrogenic systemic fibrosis (occurs in patients with renal failure usually after exposure to gadolinium-containing contrast agents) were excluded and the patient was treated, based on his typical clinical findings, for EF related to pembrolizumab. Methylprednisolone 48mg/day was initiated with rapid amelioration of the intense myalgias, oedema, and skin stiffness. After four weeks, methylprednisolone was gradually tapered to 16mg/day with relapse of symptoms. At the same time, restaging CT scans showed disease progression with new left lobe mass and the patient was placed again on chemotherapy. In rheumatology, the most common irAEs are arthritis, sicca syndrome, polymyalgia rheumatica, and myositis. Only 15 cases of checkpoint inhibitor-associated EF have been reported so far.^6^ Physicians need to be aware of this unusual and misleading diagnosis as a potential irAE. Referral to a rheumatology clinic for correct diagnosis could help to study better the irAEs following CPI treatment and to grasp the true incidence of these adverse events.
